# Transcatheter Closure of Aortic Root Rupture and Pseudoaneurysm After Surgical Implantation of Transcatheter Valve in Native Mitral Annular Calcification With Concomitant Surgically Implanted Transcatheter Aortic Valve

**DOI:** 10.1016/j.shj.2023.100205

**Published:** 2023-07-11

**Authors:** Alexander Cubberley, Rahul Sawhney, Paul Grayburn, Ambarish Gopal, Justin Schaffer, Robert L. Smith, Molly Szerlip, Srinivasa Potluri

**Affiliations:** a Department of Cardiovascular Medicine, Baylor Scott & White The Heart Hospital - Plano, Plano, Texas, USA; b Department of Interventional and Structural Cardiology, Baylor Scott & White The Heart Hospital - Plano, Plano, Texas, USA; c Department of Cardiothoracic Surgery, Baylor Scott & White The Heart Hospital - Plano, Plano, Texas, USA

**Keywords:** Aortic rupture, Paravalvular leak closure, Pseudoaneurysm, SITRAL, TAVR

## Introduction

Aortic pseudoaneurysms (PSAs) are rare complications in approximately 2% of thoracic surgery cases. They are rare after transcatheter aortic valve replacement (TAVR), but are known to be life-threatening.^1^ In patients with high-risk anatomy who are poor candidates for redo open heart surgery, percutaneous therapies offer an alternative for the management of symptoms and defects with reliable results.^2^ Most PSAs are diagnosed by echocardiography or computed tomography angiogram and can cause symptoms ranging from mild dyspnea to heart failure in rare cases. While open surgical repair is the standard therapy, percutaneous options often provide an acceptable result in high surgical risk patients.^3^^,^^4^ In high-risk patients such as those undergoing uncommon repairs, including surgical implantation of transcatheter valve in native mitral annular calcification (SITRAL) with concomitant surgically implanted TAVR who present with heart failure, a multidisciplinary, Heart Team approach with multimodality imaging provides the highest chance of a successful outcome.

## Case

### Background

**Figure 1:**
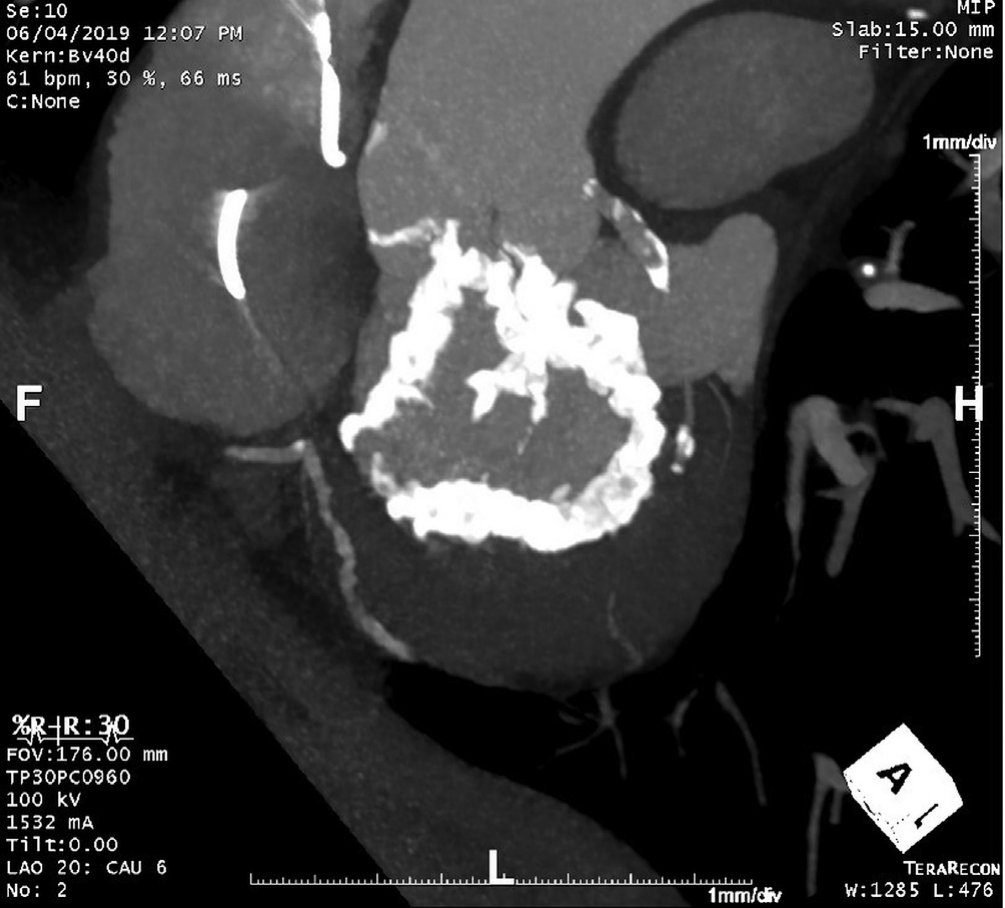
Computed tomography heart demonstrating severe circumferential mitral annular calcification extending into aortic annulus.

**Figure 2:**
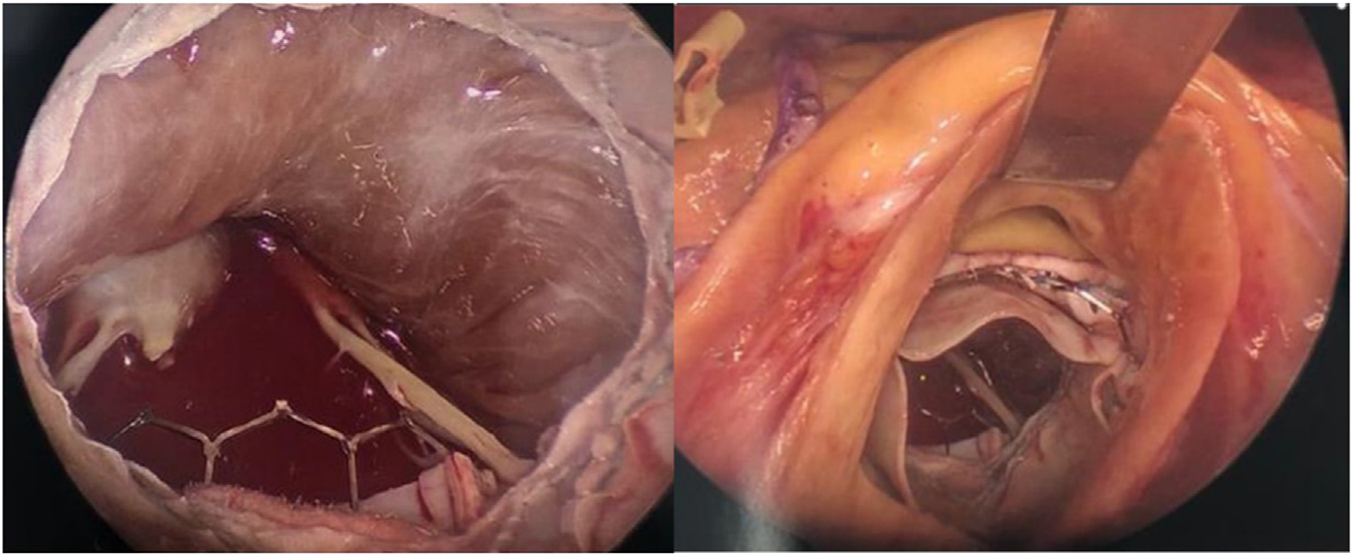
Intraoperative images of surgically implanted Sapien 3 transcatheter valve in mitral position (SITRAL) from below the aortic valve (left). SITRAL and Sapien 3 transcatheter valve in the aortic position viewed through aortotomy.

A 74-year-old male presented approximately 6 weeks after open heart surgery with worsening dyspnea on exertion, lower extremity edema, and orthopnea concerning for new-onset heart failure. He had a history of severe aortic stenosis, coronary artery disease, severe mitral valve calcification, severe mitral regurgitation, hypertension, chronic kidney disease, heart failure with preserved ejection fraction, and sinus node dysfunction with placement of a dual chamber permanent pacemaker. SITRAL-TAVR was planned due to significant mitral valve calcification extending across the aortomitral curtain and into the aortic annulus. (Figure 1) The patient underwent sternotomy with 3-vessel coronary artery bypass grafting and surgical implantation of a 29 mm Sapien 3 transcatheter valve (Edwards Lifesciences) in native mitral annular calcification with resection of the A2 mitral valve leaflet (SITRAL). The patient also underwent a 29 mm Sapien 3 transcatheter valve (Edwards Lifesciences) in the aortic position under direct visualization (Figure 2), since a sutureless valve such as the Perceval aortic valve was not immediately available at the time of the surgery. A balloon expandable 29 mm Sapien S3 was utilized in the aortic position given our medical center’s extensive experience with direct aortic placement. This was the only balloon expandable transcatheter valve available to our institution at the time of surgery. Self-expanding transcatheter valves have not been tried previously in this procedure due to concern for migration.

**Figure 3:**
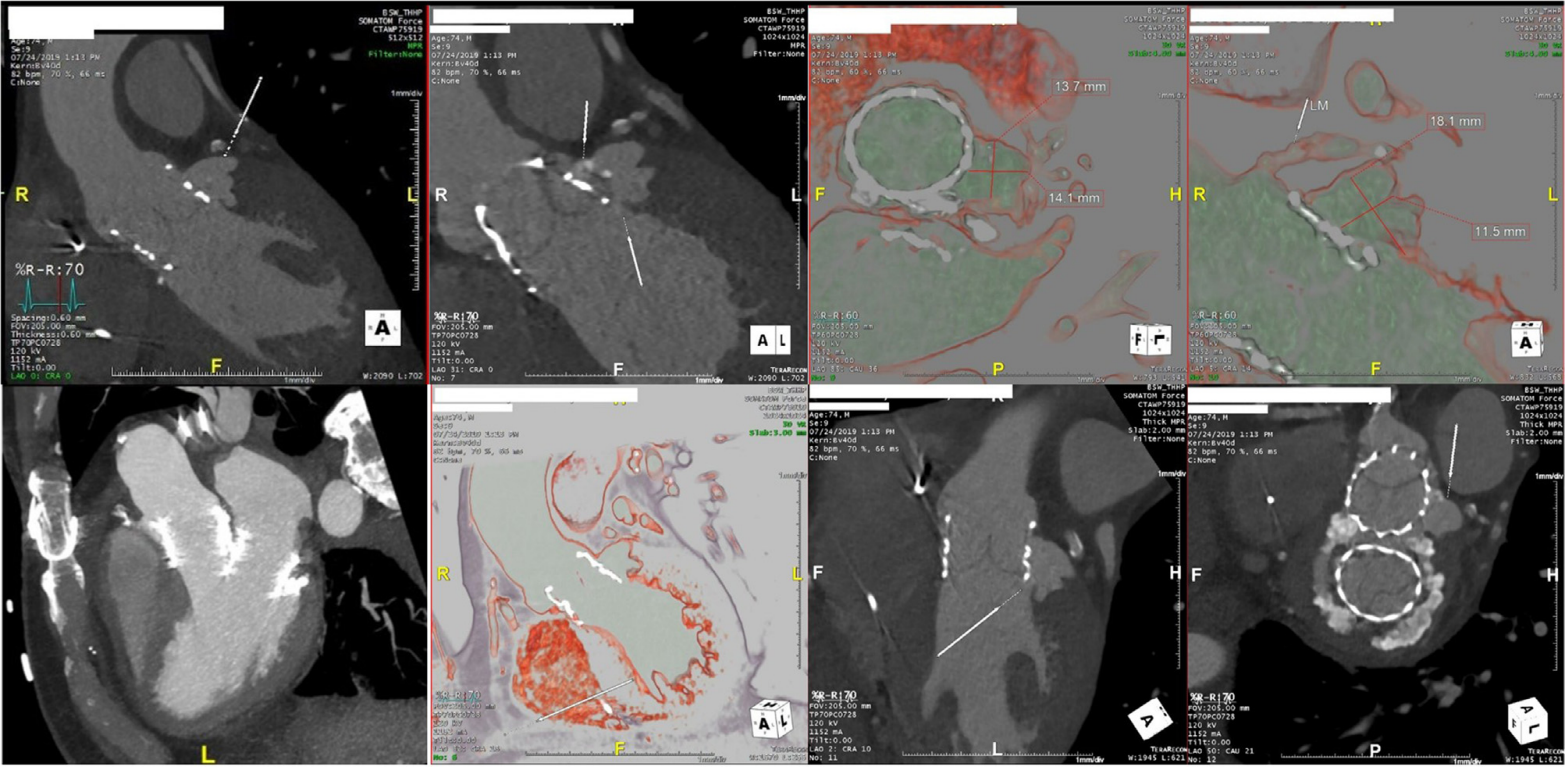
Computed tomography heart after initial surgical repair of the mitral regurgitation and aortic stenosis with Sapien 3 valve in the mitral and aortic position. Aortic annular rupture with significant aorta to left ventricle pseudoaneurysm adjacent to Sapien valve in the aorta (white arrows).

Transesophageal echocardiogram at the time of readmission demonstrated a significant paravalvular leak. Four D-CT Heart was performed which demonstrated an aortic root rupture with aorta to left ventricle (LV) PSA in the anterolateral annulus (Figure 3). Due to the complexity of this patient, he was presented at our high-risk surgical conference. Given his high mortality risk of reoperation and comorbidities, a minimally invasive intervention was elected to close the PSA.

### Intervention

**Figure 4:**
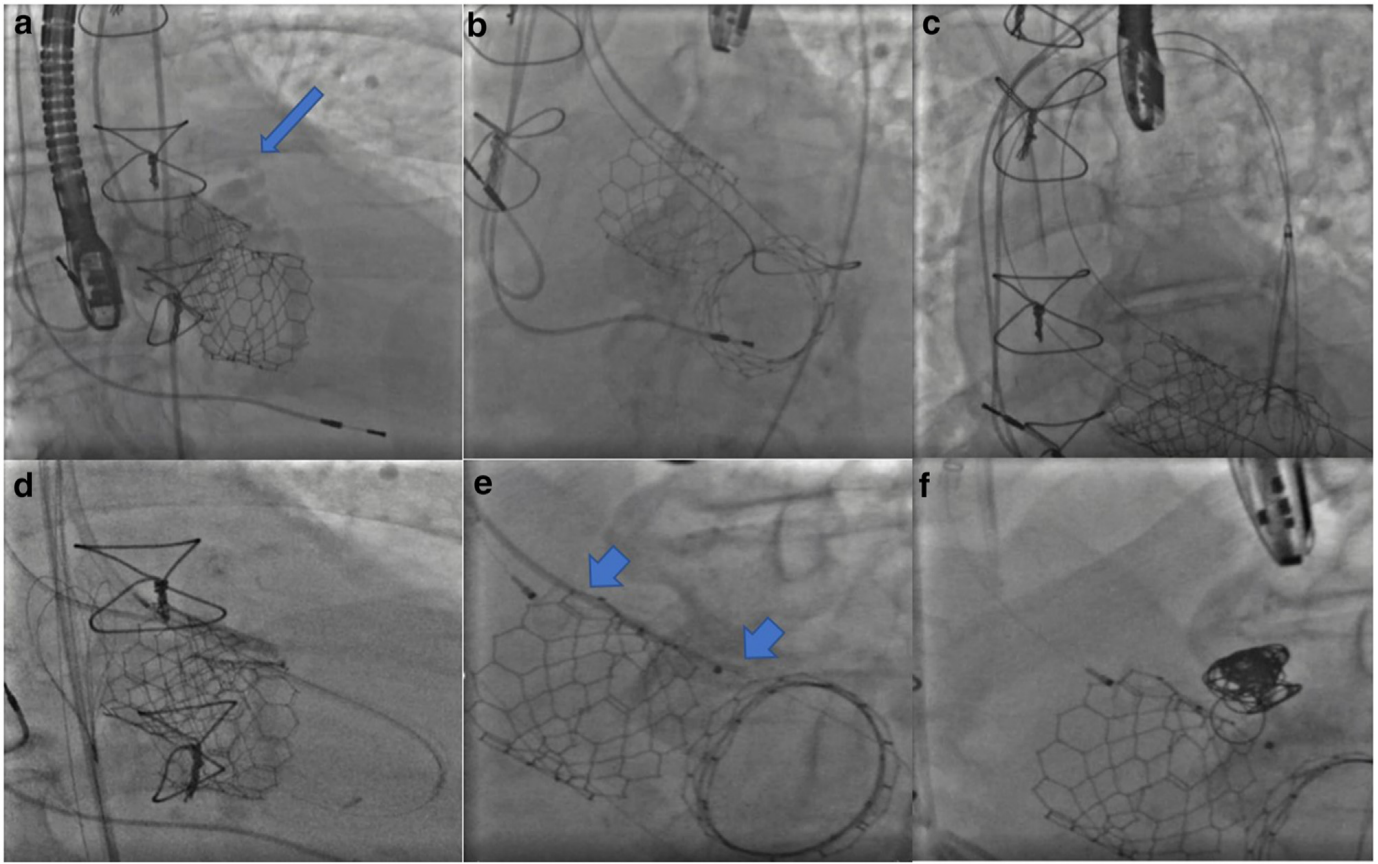
**Intervention on pseudoaneurysm.** Angiogram demonstrating aortic rupture and contrast through the pseudoaneurysm (arrow) to LV around the aortic position Sapien valve. (a) Safety wire from aorta through the pseudoaneurysm into LV, back through the center of the aortic position Sapien valve in ascending aorta (b). Safety wire snared to descending aorta (c). Microcatheter in pseudoaneurysm with placement of Amplatzer Plug (arrow) from aorta through the pseudoaneurysm into LV (d and e). Amplatzer Plug (Abbott) and distal coils (Terumo) in pseudoaneurysm (f). Abbreviation: LV, left ventricle.

**Figure 5:**
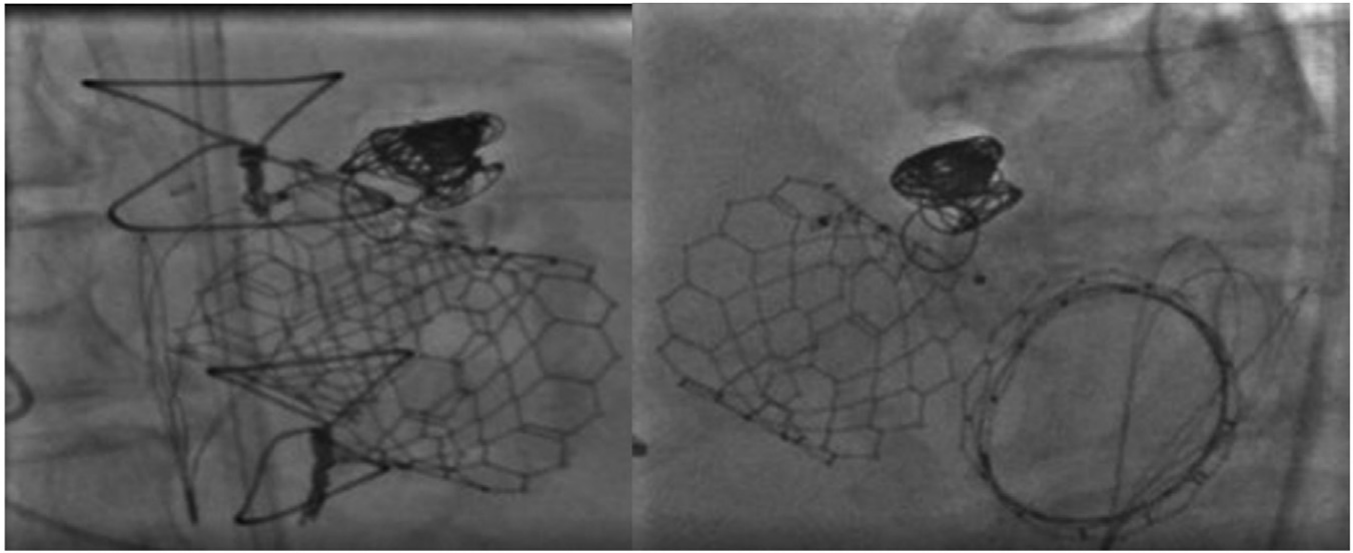
Final result after intervention: Amplatzer Plug (Abbot) and distal coils (Terumo) in the pseudoaneurysm adjacent to the aortic position Sapien transcatheter valve (Edwards).

**Figure 6:**
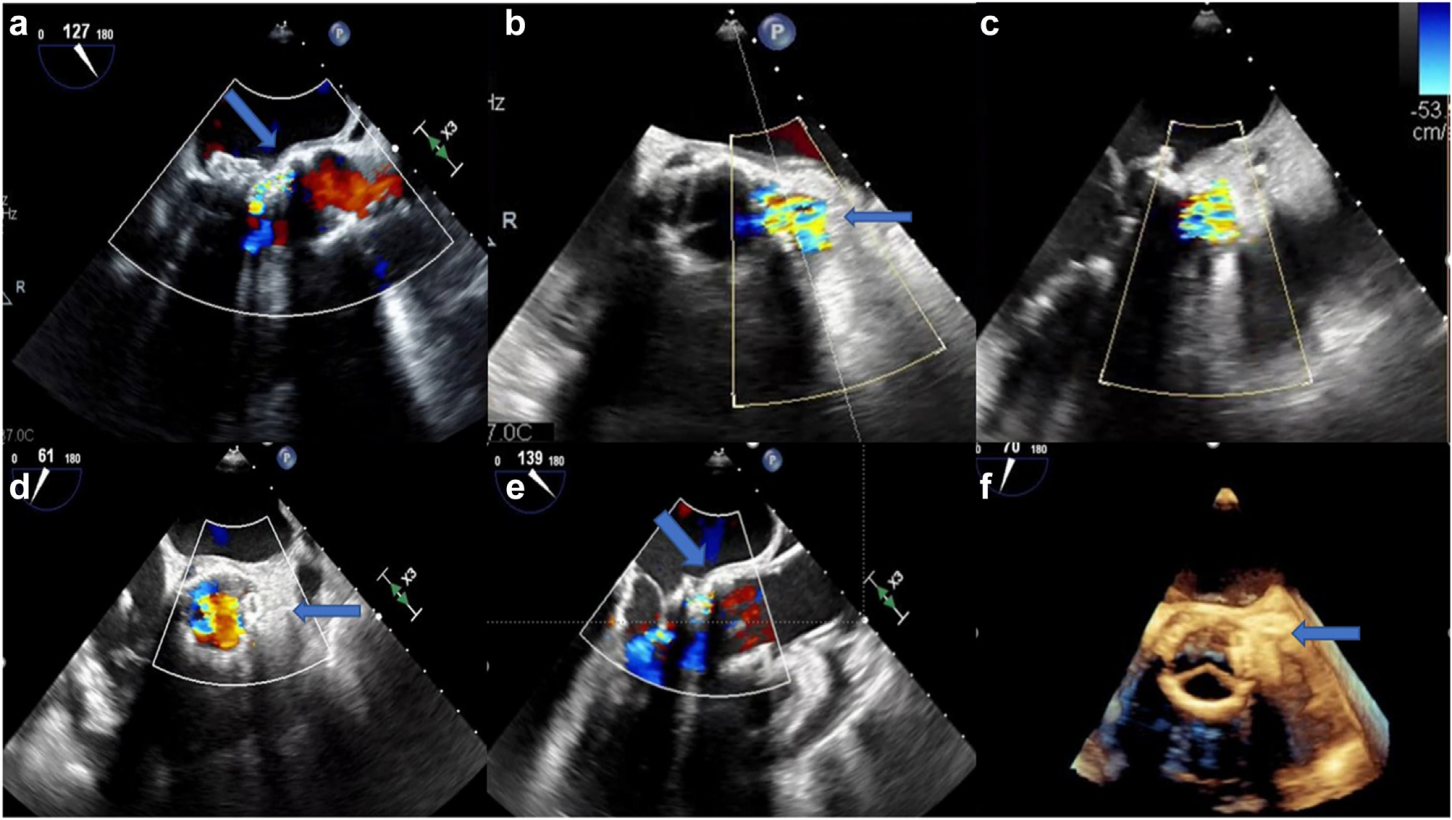
TEE 6 weeks after index procedure (SITRAL-TAVR) demonstrating pseudoaneurysm from aorta to LV adjacent to aortic Sapien transcatheter valve (arrow) (a-c). Postintervention TEE demonstrating Amplatzer Plug (arrow) and coiling of pseudoaneurysm with reduced flow around Sapien transcatheter valve (d-f). Abbreviations: TAVR, transcatheter aortic valve replacement; TEE, transesophageal echocardiogram.

The patient was taken to the Cath lab where three sheaths were placed for access, a 12 French (Fr.) DrySeal (Gore) in his right common femoral artery (CFA), an 8 Fr. sheath in his left CFA, and a 5 Fr. venous sheath in his left common femoral vein. From the R-CFA, repeat imaging was performed with a 6 Fr. JR-4 catheter. Under transesophageal echocardiogram (TEE) guidance, using a 5Fr. JR4 telescoped through the 6Fr. AL1 guide, an angled glide wire (0.035'') was easily advanced into the LV through the PSA and back into ascending aorta through the aortic Sapien valve and snared into the descending from the left CFA. JR4 was removed and a Steelcore (0.018”) Guidewire (Abbott) was inserted in the guide, advanced into the LV, and the guide was removed. A Progreat Microcatheter (Terumo) was advanced over the Steelcore wire and parked in the PSA to later use it to coil after plugging of the paravalvular leak. The Steelcore wire was then removed and reinserted through the reinserted AL1 guide into the descending aorta and used as a safety wire. Now there were two wires (glide wire and Steelcore) in the descending aorta and Progreat Microcatheter in the PSA through the 12 Fr. DrySeal sheath (Gore). A 6Fr. Destination Peripheral Guiding sheath (Terumo) was advanced into the DrySeal (Gore) over the glide wire and advanced into the LV. The Glidewire was removed and a 12 mm Amplatzer Vascular Plug II (Abbott) deployed in the paravalvular leak. After satisfactory positioning of the vascular plug, coiling of the PSA was performed with four coils: one Azur (Terumo) 14 mm × 24 cm Framing Coil, two Azur 18 C × 8 mm × 28 cm coils, and one Azur 18 C × 7 mm × 24 cm coil were deployed successfully. Coiling was done to occlude the lumen of the pseudoaneurysm given its large neck diameter that was not fully filled by the Amplatzer Vascular Plug. After TEE and fluoroscopy confirmation of stability of coils and positioning of vascular plug, the vascular plug was deployed (Figures 4 and 5). Decreased flow through the PSA was confirmed on TEE (Figure 6).

### Follow-Up

**Figure 7:**
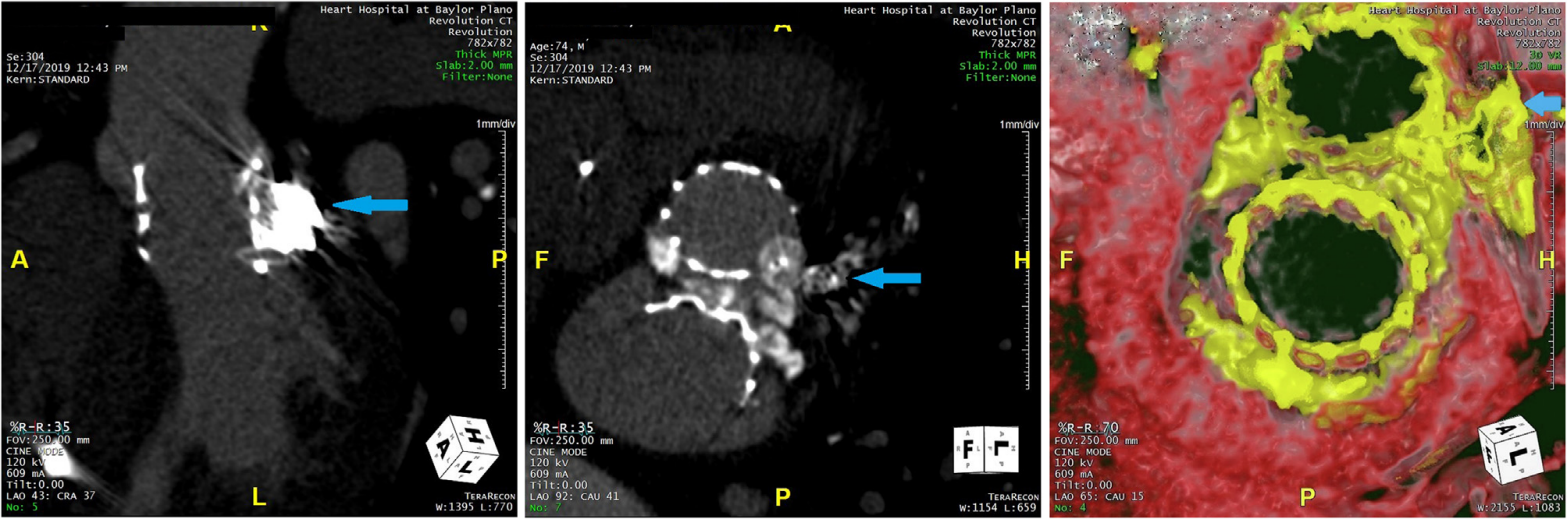
Follow-up CT heart 6 months after intervention showing Sapien valves with occlusion of pseudoaneurysm (arrows) by Amplatzer Plug and coils (left, middle). 3 dimensional CT reconstruction showing Sapien valves (aortic valve-top, mitral valve-bottom) with closure of pseudoaneurysm (arrow, right). Abbreviation: CT, computed tomography.

Patient was discharged 2 ​days after the procedure with significant improvement in his shortness of breath. Echocardiogram at follow-up visit, 1-month postintervention, demonstrated reduced aortic regurgitation from severe to trace. Our patient still demonstrated moderate dyspnea and fatigue on exertion. His medical therapy, including beta-blocker, was further adjusted at this time. Patient was seen in follow-up 6 months and 1 year after the procedure with improvement in symptoms, only mild residual dyspnea without orthopnea, paroxysmal nocturnal dyspnea, or edema. A 4 dimensional computer tomography of the heart 6 months after closure demonstrated no residual PSA (Figure 7). Repeat echocardiogram at 6 months and 1 year demonstrated mild paravalvular aortic regurgitation. Our patient will continue to follow-up yearly in our combined surgery and Transcatheter Valve Clinic.

## Discussion

In patients with significant mitral annular calcification and mitral-aortic curtain calcification with aortic stenosis and mitral valve disease, valve replacement by SITRAL and TAVR provide an alternative to traditional valve replacement. A rare complication of PSA formation after valve implantation can occasionally lead to heart failure symptoms but also remain at risk for rupture in the future. Transesophageal echo was utilized early in the diagnostic work as there was high suspicion that the patient's heart failure was valve related. Transthoracic imaging would not provide appropriate visualization of the surgically implanted mitral valve. While previous centers have closed aorta to LV PSA with Amplatzer Plugs, this is the first report of percutaneous closure of an aortic root rupture and PSA with plugging and coils in a patient who previously underwent SITRAL-TAVR.^5^ Collaboration with the Heart Team (cardiothoracic surgery, interventional cardiology, heart failure and advanced cardiac imaging) was instrumental in the success of this procedure. TEE and four-dimensional CT play an essential role in the accurate diagnosis of PSA and guidance of structural heart disease procedural planning.

## Consent Statement

The patient has consented to the publication of this report and accompanying images.

## Funding

The authors have no funding to report.

## Disclosure Statement

The authors report no conflict of interest.
